# Elevated CK-MB levels are associated with adverse clinical outcomes in acute pancreatitis: a propensity score-matched study

**DOI:** 10.3389/fmed.2023.1256804

**Published:** 2023-09-08

**Authors:** Xiao-Ting Jiang, Ling Ding, Xin Huang, Yu-Peng Lei, Hua-Jing Ke, Hui-Fang Xiong, Ling-Yu Luo, Wen-Hua He, Liang Xia, Nong-Hua Lu, Yin Zhu

**Affiliations:** Department of Gastroenterology, Digestive Disease Hospital, The First Affiliated Hospital of Nanchang University, Nanchang, Jiangxi, China

**Keywords:** acute pancreatitis, cardiac injury, adverse clinical outcomes, creatine kinase-myocardial band, propensity score-matching methods

## Abstract

**Aim:**

Cardiac injury, reflected by the measured concentrations of chemicals released from injured cardiac muscle, is common in acute pancreatitis (AP). However, there is no adequate evidence assessing the impact of cardiac injury on AP-related outcomes. Creatine kinase-myocardial band (CK-MB) mainly exists in the myocardium. Therefore, we sought to evaluate the relationship between the increase in CK-MB and the adverse clinical outcomes of AP.

**Methods:**

This propensity score-matched study analyzed AP patients admitted to the Department of Gastroenterology in the First Affiliated Hospital of Nanchang University from June 2017 to July 2022. Propensity score matching and multivariate logistic regression analysis were used to explore the relationship between CK-MB elevation and AP outcome variables.

**Results:**

A total of 5,944 patients were screened for eligibility, of whom 4,802 were ultimately enrolled. Overall, 896 (18.66%) of AP patients had elevated (>24 U/ml) CK-MB levels, and 895 (99.89%) were paired with controls using propensity score matching. The propensity score-matched cohort analysis demonstrated that mortality (OR, 5.87; 95% CI, 3.89–8.84; *P* < 0.001), severe acute pancreatitis (SAP) (OR, 2.74; 95% CI, 2.23–3.35; *P* < 0.001), and infected necrotizing pancreatitis (INP) (OR, 3.40; 95% CI, 2.34–4.94; *P* < 0.001) were more frequent in the elevated CK-MB (>24 U/ml) group than in the normal CK-MB (≤ 24 U/ml) group. Using the multivariate logistic regression analysis, elevated CK-MB levels were independently associated with increased mortality (OR, 2.753, 95% CI, 2.095–3.617, *P* < 0.001), SAP incidence (OR, 2.223, CI, 1.870–2.643, *P* < 0.001), and INP incidence (OR, 1.913, 95% CI, 1.467–2.494, *P* < 0.001). CK-MB elevation was an independent risk factor for adverse clinical outcomes in AP patients.

**Conclusion:**

CK-MB elevation was significantly related to adverse outcomes in AP patients, which makes it a potentially useful laboratory parameter for predicting adverse clinical outcomes of AP.

## Introduction

Acute pancreatitis (AP) is an acute inflammatory disease. Most patients with AP can be discharged within 1 week after conservative treatment, but 20% develop severe acute pancreatitis (SAP). The large-scale infiltration of inflammatory cells will eventually trigger systemic inflammatory response syndrome (SIRS) or even multiple organ dysfunction syndrome (MODS), which have high mortality rates of 20–40% (1, 2).

Acute pancreatitis is a systemic inflammatory disorder. During the acute course, the heart is damaged by various inflammatory factors. Many experiments in animal models of SAP have observed pathological ultrastructural damage to the heart, including myocardial edema, myocardial hypertrophy, myocardial apoptosis, myocardial fibrosis, and excessive contraction of collagen fibers, accompanied by cardiac dysfunction and the elevation of cardiac-related enzymes (3–5). Prasada et al. (6) research showed that 40% of patients with AP had electrocardiogram (ECG) abnormalities during the disease and 85% of patients presented with ST-segment depression with T-wave inversion. Cardiac markers such as troponins I and T, creatine kinase (CK), creatine kinase-myocardial band (CK-MB) isoenzyme, and lactate dehydrogenase (LDH) are released into the circulation during acute cardiac dysfunction; CK-MB mainly exists in myocardial cells and has high sensitivity and specificity as an indicator of myocardial injury (7).

However, the results remained inconsistent regarding the impact of elevated CK-MB on the adverse outcomes of AP. A previous study showed that CK-MB might be an effective biomarker to predict the occurrence of SAP and the development of organ failure in the early stages of AP (8). In contrast, Barassi et al. (9) research showed that CK-MB was not related to the severity of AP. Therefore, we conducted a single-center, retrospective, large-sample cohort study. Propensity score matching analysis and multivariate logistic regression analysis were used to confirm the effect of serum CK-MB levels on clinical outcomes and the healthcare utilization of patients with AP.

## Methods

### Study design and participants

This was a propensity score-matched study. We analyzed the data of patients with AP admitted to the Department of Gastroenterology of the First Affiliated Hospital of Nanchang University from June 2017 to July 2022. These data were from a prospectively maintained database of the Department of Gastroenterology of the First Affiliated Hospital of Nanchang University, which is updated and maintained by specialized personnel and contains patient data on demographics, medical records, laboratory indicators, and follow-up information of patients with pancreatitis. Patients in the database who had been diagnosed with AP were included in the study.

The exclusion criteria for patients were as follows: (1) a history of chronic pancreatitis; (2) no examination of CK-MB; and (3) diseases known to increase the level of myocardial injury markers (CK-MB, CK, etc.), such as chronic heart disease, coronary atherosclerotic heart disease, congestive heart failure, severe valvular disease and/or cardiomyopathy, chronic obstructive pulmonary disease (COPD), chronic renal failure, cancer (including pancreatic cancer), immune deficiency disease, and central nervous system disease (such as meningitis, brain abscesses, and cerebral hemorrhage). This study was approved by the Ethics Committee of the First Affiliated Hospital of Nanchang University. As the study was retrospective, the Ethics Committee waived the requirement of written informed consent.

### Serum measurement and data collection

Cardiac marker data were collected within 24 h of each patient's admission. Patients with AP admitted at different onset times received blood collected by designated personnel. All tests were completed within 24 h and measured using the enzyme rate immunosuppression method (instrument: Hitachi 7200). The normal reference ranges were 0–24 U/ml for CK-MB, 40–200 U/ml for CK, 120–250 U/ml for lactate dehydrogenase (LDH), and 3–35 U/ml for aspartate aminotransferase (AST); if it was more than 24 U/ml, CK-MB was considered to be elevated. Based on the maximum values of CK-MB, AST, LDH, and CK, all enrolled patients were divided into elevated and normal groups. General information such as gender, age, body mass index (BMI), etiology, time from onset to admission, comorbidities (hypertension and diabetes mellitus), and smoking and alcohol history were included.

### Clinical outcomes

The adverse clinical outcomes observed in this study included mortality, severe AP (SAP), and infected necrotizing pancreatitis (INP), which showed a more serious condition. According to the 2012 revised Atlanta guidelines, SAP is characterized by persistent (>48 h) organ failure, including the respiratory, cardiovascular, or renal systems. INP was confirmed by extraluminal gas in the pancreatic and/or peripancreatic tissues on contrast-enhanced computed tomography (CECT) or positive bacteria and/or fungi on Gram stain or culture of pancreatic and/or peripancreatic tissues obtained by percutaneous image-guided fine-needle aspiration or from the first drainage procedure or the first necrosectomy (10). The length of hospital stays, the length of intensive care unit (ICU) stays, and total hospital costs were included, all of which indicated the severity of AP.

### Statistical analysis

Quantitative variables are presented as medians (interquartile ranges) and were analyzed using the Mann–Whitney *U*-test. Categorical variables are reported as absolute numbers and proportions and were tested by the chi-square test. Factors in unadjusted models (*P* < 0.05) were included in the multivariate logistic regression analysis (stepwise, sls =0.10, sle = 0.05) to identify the risk factors independently associated with adverse clinical outcomes. All results are presented as odds ratios (ORs) and 95% confidence intervals (CIs). A two-tailed *P* < 0.05 was considered statistically significant. The data were analyzed using IBM SPSS Statistics 25.

The multivariate logistic regression model was used to calculate the propensity scores of each subject, with the increase in CK-MB as the dependent variable and the patient demographics, admission time, and comorbidities as the independent variables. Patients with elevated CK-MB levels and patients with normal CK-MB levels were matched 1:1 using a greedy matching algorithm with a caliper of 0.2 times the SD of the propensity scores.

## Results

### Patient characteristics

**Figure 1:**
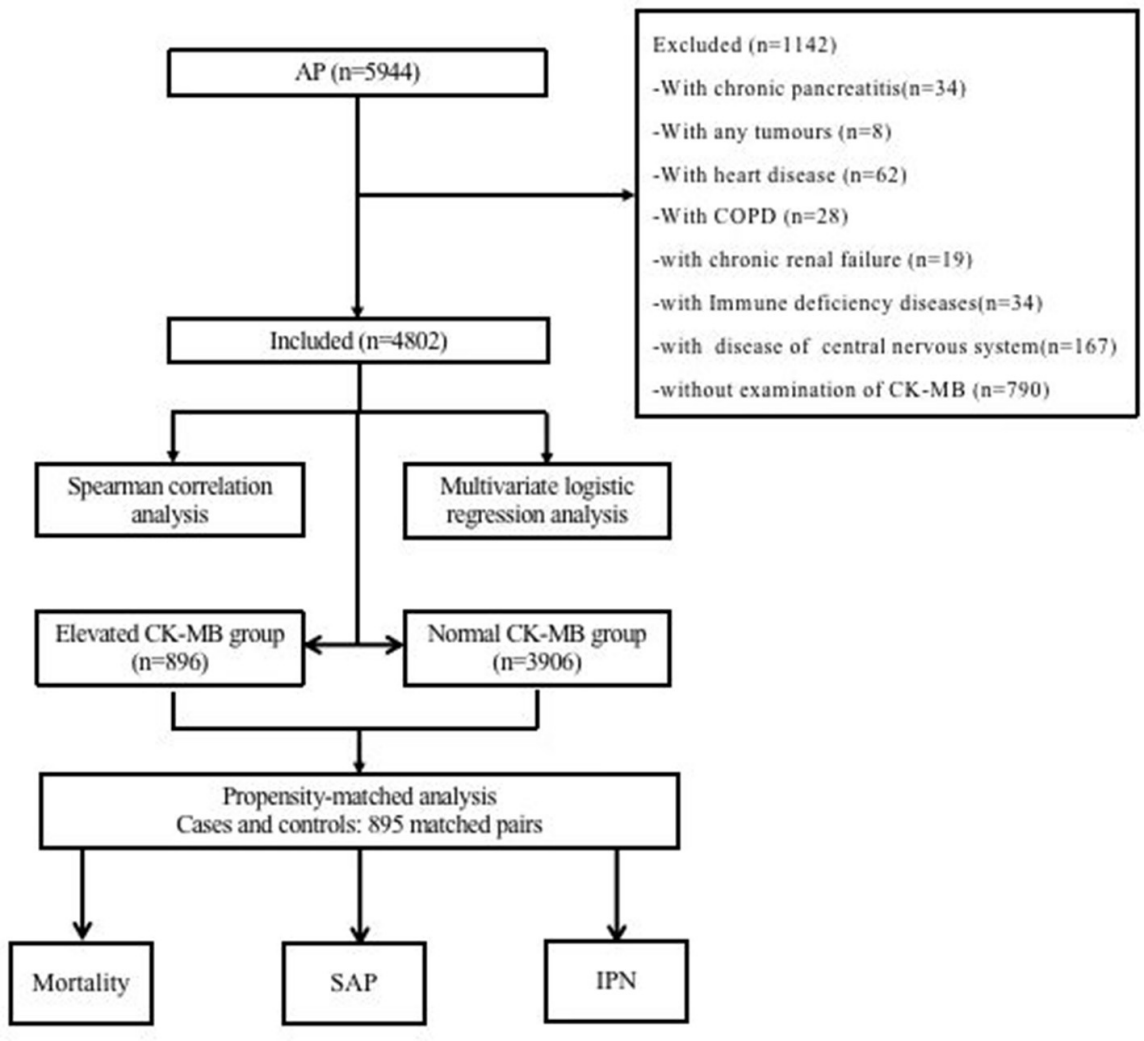
Flow diagram of patient inclusion and exclusion. AP, acute pancreatitis; COPD, chronic obstructive pulmonary disease; CK-MB, creatine kinase-myocardial band; SAP, severe acute pancreatitis; IPN, infected pancreatic necrosis.

We screened 5,944 patients with AP from the database, and 4,802 patients were included in this study, as shown in Figure 1. Among these subjects, 896 (18.66%) patients were in the CK-MB elevated group (>24 U/ml) and 3,906 (81.34%) were in the normal group.

Among these patients, 62.24% were men, with a median age of 49 years. The median time from onset to admission was 3 (1, 6) days, with 57.25% of patients being admitted within 3 days after the onset, which showed that most patients were admitted in the early course of the disease. Among them, acute biliary pancreatitis accounted for the majority, up to 45.48% (2,184/4,802). A total of 1,107 patients had SAP, accounting for 23.05% according to the 2012 Atlanta guidelines.

**Figure 2:**
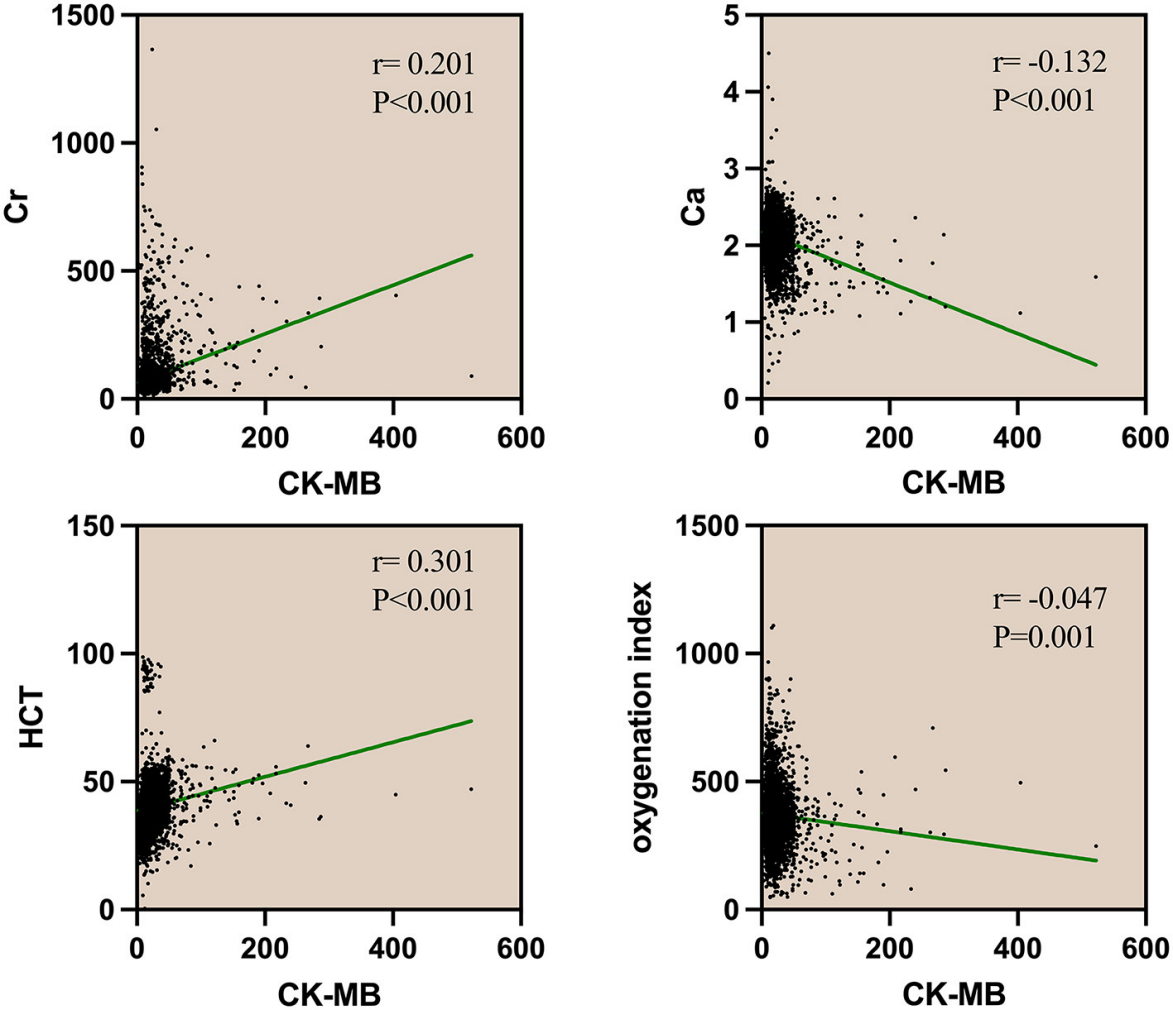
Spearman correlation analysis of CK and CK-MB with Cr, Ca, HCT, and oxygenation index. Cr, serum creatinine; Ca, serum calcium; HCT, hematocrit; CK, creatine kinase; CK-MB, creatine kinase-myocardial band.

Many prognostic scoring systems have shown that an increase in hematocrit and creatinine and a decrease in blood calcium and oxygenation index are associated with adverse clinical outcomes in AP (11). In the analysis of the correlation between CK-MB and blood calcium (Ca), creatinine (Cr), oxygenation index (OI), or hematocrit (HCT) within 24 h of admission, the correlation analysis revealed that serum creatinine and hematocrit showed a positive correlation with the level of CK-MB (*r* = 0.201, *r* = 0.301). By comparison, serum calcium and the oxygenation index showed a negative correlation (*r* = −0.132, *r* = −0.047). The four biochemical markers showed a significant correlation with CK-MB (*P* < 0.001), indicating a possible correlation between elevated CK-MB levels and acute pancreatitis (Figure 2).

### Propensity-matched cohort analysis of adverse clinical outcomes

To study the impact of CK-MB elevation on the outcomes of AP, we established an unweighted matched cohort for CK-MB using propensity-matched cohort analysis (Figure 1). A total of 4,802 AP patients were divided into a CK-MB elevated group and a CK-MB normal group according to the level of CK-MB. Finally, 895 pairs were matched for a 1:1 ratio. There were no significant differences in baseline characteristics, hospital admission time, personal preferences, or comorbidities between the reconstruction groups. After matching, conditional logistic regression analysis for the AP-related outcomes continued to demonstrate that the increased CK-MB group was associated with adverse clinical outcomes, including mortality (OR, 5.87; 95% CI, 3.89–8.84; *P* < 0.001), SAP (OR, 2.74; 95% CI, 2.23–3.35; *P* < 0.001), and INP (OR, 3.40; 95% CI, 2.34–4.94; *P* < 0.001). We also found that the elevated CK-MB group not only prolonged the length of hospital stay (OR, 6.21; 95% CI, 4.58–7.84; *P* < 0.001) but also contributed to the length of stay in the ICU (OR, 5.35; 95% CI, 4.33–6.38; *P* < 0.001). Analysis of hospital charges revealed that patients with elevated CK-MB levels had higher total hospital expenses (*P* < 0.001). More details are shown in Tables 1, 2.

**Table 1:** Baseline characteristics before and after a 1:1 propensity score-matched analysis with elevated CK-MB levels (>24 U/ml) and normal CK-MB levels ( ≤ 24 U/ml).

**Characteristics**	**Before matching**		***P***	**After matching**		***P*-value**
	**Elevated CK-MB (*****n** =* **896)**	**No elevated CK-MB** **(*****n** =* **3,906)**		**Elevated CK-MB** **(*****n** =* **895)**	**No elevated CK-MB (*****n** =* **895)**	
Male, *n* (%)	612 (68.30)	2,377 (60.86)	0.001	611 (68.16)	600 (67.04)	0.578
Age, years	47 (36,60)	50 (37,62)	0.004	47 (36,60)	47 (36,60)	0.844
BMI, kg/m^2^	24.22 (22.42, 26.95)	23.53 (21.45, 25.71)	0.001	24.22 (22.41, 26.95)	24.22 (22.04, 26.63)	0.315
Admission time after AP	2 (1.00, 4.00)	3 (2.00, 7.00)	0.001	2 (1.00, 4.00)	2 (1.00, 4.00)	0.092
Onset, days						
Etiology, *n* (%)			0.001			0.079
Biliary	314 (35.04)	1,870 (47.88)		314 (35.08)	345 (38.55)	
Alcoholic	49 (5.47)	209 (5.35)		49 (5.47)	45 (5.03)	
Hyperlipidemia	379 (42.30)	1,104 (28.26)		379 (42.35)	349 (38.99)	
Others	81 (9.04)	509 (13.03)		81 (9.05)	103 (11.51)	
Mixed	73 (8.15)	214 (5.48)		72 (8.04)	53 (5.92)	
Smoking history, *n* (%)	302 (33.71)	1,092 (27.96)	0.001	301 (33.63)	274 (30.61)	0.172
Drinking history, *n* (%)	317 (35.38)	248 (24.87)	0.001	316 (35.31)	292 (32.63)	0.231
Hypertension, *n* (%)	188 (20.98)	683 (17.49)	0.014	187 (20.89)	193 (21.56)	0.729
Diabetes mellitus, *n* (%)	135 (15.07)	447 (11.44)	0.003	135 (15.08)	126 (14.08)	0.547

BMI, body mass index; AP, acute pancreatitis; CK-MB, creatine kinase-myocardial band. Quantitative variables are presented as medians (interquartile ranges) and were analyzed using the Mann–Whitney U-test. Categorical variables are reported as absolute numbers and proportions and were tested using a chi-square test.

**Table 2:** Differences in adverse clinical outcomes and healthcare utilization in a 1:1 propensity score-matched analysis of 895 matched pairs of patients with elevated CK-MB levels (>24 U/ml) and normal CK-MB levels ( ≤ 24 U/ml).

	**Elevated CK-MB group (*n =* 895)**	**No elevated CK-MB group (*n =* 895)**	**OR/average difference**	**95% CI**	***P*-value**
Mortality	147 (16.42%)	29 (3.24%)	5.87	(3.89, 8.84)	<0.001
SAP	407 (45.47%)	209 (23.35%)	2.74	(2.23, 3.35)	<0.001
IPN	120 (13.41%)	39 (4.36%)	3.40	(2.34, 4.94)	<0.001
Length of hospital stay, days	18.27 ± 20.59	12.07 ± 13.57	6.21	(4.58, 7.84)	<0.001
Length of ICU stay, days	7.37 ± 13.75	2.02 ± 7.00	5.35	(4.33, 6.38)	<0.001
Total hospitalization charges, thousand yuan	51.13 ± 280.49	95.32 ± 172.62	44.19	(23.73, 64.64)	<0.001

OR, odds ratio; CI, confidence interval; CK-MB, creatine kinase-myocardial band; SAP, severe acute pancreatitis; IPN, infected pancreatic necrosis; ICU, intensive care unit. Categorical variables are expressed in proportions and performed using conditional logistic regression analysis. Quantitative variables were expressed as mean ± standard deviation and analyzed using a paired t-test.

### Univariate and multivariate logistic regression analyses of adverse clinical outcomes

**Figure 3:**
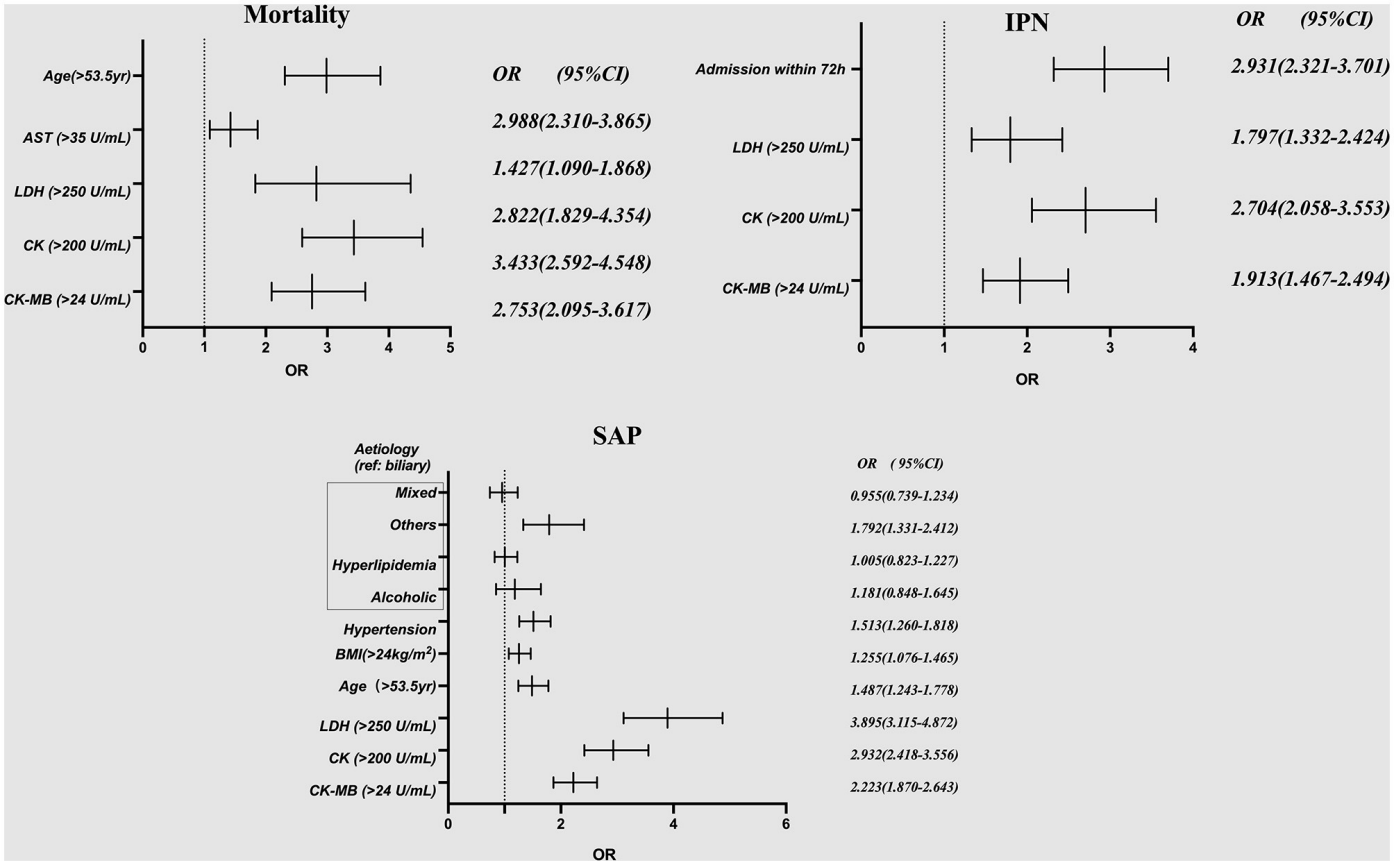
Multivariate logistic regression analysis for the association of patients' demographic characteristics, etiologies, and comorbid conditions with the risk of mortality, SAP and IPN among all 4,802 patients. AST, aspartate aminotransferase; LDH, lactate dehydrogenase; CK, creatine kinase; CK-MB, creatine kinase-myocardial band; SAP, severe acute pancreatitis; IPN, infected pancreatic necrosis.

The results of univariate analysis between the cardiac markers and adverse clinical outcomes of 4,802 unmatched patients are shown in Supplementary Tables S2–S4. The factors with *P* < 0.05 in the univariate analysis were finally included in a multivariate logistic regression analysis. Elevated CK-MB levels were independently associated with higher incidence rates of mortality (OR, 2.753; 95% CI, 2.095–3.617; *P* < 0.001), SAP (OR, 2.223; CI, 1.870–2.643; *P* < 0.001), and INP (OR, 1.913; 95% CI, 1.467–2.494; *P* < 0.001). The other three cardiac markers, CK (OR, 3.433; 95% CI, 2.592–4.548; *P* < 0.001), AST (OR, 1.427; 95% CI, 1.090–1.868; *P* < 0.001), and LDH (OR, 2.822; 95% CI, 1.829–4.354; *P* < 0.001), were independent risk factors for mortality in patients with AP. However, analysis between the AST-elevated group and the normal group revealed no differences in the prevalence of SAP or INP (*P* > 0.05) (Table 3, Figure 3). A subgroup analysis by the time of onset of AP was also conducted. We divided patients into two groups based on their admission time: in the early (within 72 h) and late (after 72 h) stages of pancreatitis. Through the univariate analysis, it was found that the elevation of CK-MB was significantly related to adverse outcomes such as mortality, SAP, and INP in patients with AP (*P* < 0.005) in two groups.

**Table 3:** Multivariate logistic regression analysis for the association of patients' demographic characteristics, etiologies, and comorbid conditions with the risk of mortality, SAP, and IPN among all 4,802 patients.

	**OR**	**95% CI**	***P*-value**
**Mortality**			
CK-MB (ref: ≤ 24 U/ml)			
>24	2.753	2.095–3.617	<0.001
CK (ref: ≤ 200 U/ml)			
>200	3.433	2.592–4.548	<0.001
LDH (ref: ≤ 250 U/ml)			
>250	2.822	1.829–4.354	<0.001
AST (ref: ≤ 35 U/ml)			
>35	1.427	1.090–1.868	0.010
Age (ref: ≤ 53.5 years)			
>53.5	2.988	2.310–3.865	<0.001
**SAP**			
CK-MB (ref: ≤ 24 U/ml)			
>24	2.223	1.870–2.643	<0.001
CK (ref: ≤ 200 U/ml)			
>200	2.932	2.418–3.556	<0.001
LDH (ref: ≤ 250 U/ml)			
>250	3.895	3.115–4.872	<0.001
Age (ref: ≤ 53.5 years)			
>53.5	1.487	1.243–1.778	<0.001
BMI (ref: ≤ 24 kg/m^2^)			
>24	1.255	1.076–1.465	0.004
Hypertension (ref: no)			
Yes	1.513	1.260–1.818	<0.001
Etiology (ref: biliary)			
Alcoholic	1.181	0.848–1.645	0.324
Hyperlipidemia	1.005	0.823–1.227	0.963
Others	1.792	1.331–2.412	<0.001
Mixed	0.955	0.739–1.234	0.724
**IPN**			
CK-MB (ref: ≤ 24 U/ml)			
>24	1.913	1.467–2.494	<0.001
CK (ref: ≤ 200 U/ml)			
>200	2.704	2.058–3.553	<0.001
LDH (ref: ≤ 250 U/ml)			
>250	1.797	1.332–2.424	<0.001
Admission within 72 h after AP onset	2.931	2.321–3.701	<0.001

OR, odds ratio; CI, confidence interval; SAP, severe acute pancreatitis; IPN, infected pancreatic necrosis; BMI, body mass index; AP, acute pancreatitis; AST, aspartate transaminase; LDH, lactate dehydrogenase; CK, cretin kinase; CK-MB, creatine kinase-myocardial band. Factors associated with adverse clinical outcomes in univariate analysis (*P* < 0.05) were included in the multivariable models, and only results with *P* < 0.05 in multivariate regression analysis were listed above.

## Discussion

This was a large sample, propensity score-matched study to explore the relationship between cardiac markers, especially CK-MB, and adverse clinical outcomes of AP. Cardiac injury is one of the most common types of organ damage in AP. In our study, the total occurrence rates of elevated CK-MB (>24 U/ml), AST (>35 U/ml), LDH (>250 U/ml), and CK (>200 U/ml) were 1,896 (18.66%), 2,154 (44.86%), 3,379 (70.37%), and 615 (12.81%), respectively. The propensity score matching analysis and the multivariate logistic regression analysis suggested that CK-MB increased the risk of adverse clinical outcomes in patients with AP, including mortality, SAP, and INP, and accounted for greater healthcare utilization.

In AP, inflammatory reactions in the pancreas lead to multiple organ damage throughout the body, and the heart is often one of the affected organs. The 2012 revised Atlanta guidelines defined cardiovascular failure in AP as a systolic blood pressure of <90 mmHg, which has no response to liquid treatment (10). However, there is a lack of specific definitional criteria for AP-associated cardiac injury. Creatine phosphokinase is involved in the process of ATP formation. It is abundant in the cytoplasm and mitochondria of cells that need a large amount of ATP, such as brain, skeletal muscle, and heart cells. The isoenzyme CK-MB mainly exists in the myocardium (12). A prospective study found that 27.7% of patients with AP within 24 h of onset had an increase in the level of CK-MB (more than twice the normal value) (6). The incidence was much higher than in our study (18.66%). However, ~70% of the patients included in the study had moderately severe or severe pancreatitis, which also revealed that CK-MB was related to the deterioration of patients with AP. In our study, moderately severe or severe pancreatitis accounted for 58.45%. In addition to the elevation of CK-MB, other serum cardiac indicators (CIs), such as N-pro-BNP, troponin I and T, serum hydroxybutyrate dehydrogenase, and CK and LDH, have been reported in patients with AP (9, 13–15). The cardiac manifestations of AP present with hemodynamic changes, EKG changes, and pericardial changes (15). Acute myocardial infarction also occurs in SAP, which can be fatal (16).

Mao conducted a retrospective study on the levels of three cardiac indicators (CIs) within 48 h of admission in 2020 and found that the elevated level of CK-MB can be used as an effective biomarker to predict the occurrence of SAP and the development of organ failure (OF) at admission (8). Similarly, in our large-sample study, CK-MB, as an independent risk factor for adverse clinical outcomes in patients with AP, was of great significance for the early prediction of severity. Barassi's research showed a negative result. In this study, 37 cases of AP within 2 days after the onset of AP were included in the observation, and serum cardiac indicators (CIs) [troponin T (cTnT), CK-MB, and serum B-type natriuretic (NT-pro-BNP)] 1–3 days after admission were detected. The results showed that CK was related to SAP, while cTnT, CK-MB, and other indicators were not related to the severity of AP (*P* > 0.05) (9). This may be due to the small sample size included in this study and the use of univariate analysis. Moreover, they found that 40.0% of patients with SAP had an abnormally high CK value, but 9.1% of patients had MAP (*P* = 0.042), which is consistent with the results of our study (OR, 2.932; 95% CI, 2.418–3.556; P 0.001). Myocardial injury in patients with AP is a process of inflammatory injury induced by multiple factors, multiple links, and multiple organs. Various risk factors cause damage to pancreatic acinar cells, leading to the release of pancreatic hydrolase. These enzymes can overactivate inflammatory response cells, resulting in the accumulation of a large number of inflammatory factors, such as tumor necrosis factor-alpha and interleukin-10 beta, and enhance their autocrine (17, 18). Meanwhile, the accumulation of ROS can increase oxidative stress by activating the MAPK pathway (19). On the other hand, because intestinal barrier function is damaged, enterotoxins enter the bloodstream. These inflammatory cytokines and toxins simultaneously cause multiple organ damage throughout the body, ultimately resulting in cardiac injury in AP.

The correlation analysis of CK-MB and some laboratory parameters reflecting intravascular volume status, pancreatic tissue perfusion, necrosis, and even organ failure development showed that with the increase in CK-MB, serum creatinine and hematocrit also increased. Recently, the harmless AP score (HAPS) (no rebound tenderness and/or guarding, normal hematocrit level, and normal serum creatinine level) was shown not to be severe within 30 min of admission (20), which is consistent with the conclusion of this study. When there was an increase in CK-MB, AP was more likely to develop into a severe disease, accompanied by an increase in hematocrit and creatinine. At present, there are a large number of multifactor scoring systems and biochemical markers to predict the severity of AP. This study also shows that CK-MB has some advantages. In the future, more prospective and follow-up studies are needed to further explore the relationship between CK-MB and AP.

Our study still has some shortcomings. First, although our large sample provided a reasonable estimation of the impact of serum cardiac indicators (CIs) in AP, this study was a single-center study with limited representativeness of the results. Second, this study was retrospective. Third, CK-MB reported that the peak level appeared in the early stage of SAP and then decreased (9). We did not continuously observe the dynamic change in the CIs over the whole course of patients' admission. However, in our study, the median time from onset to admission was 3 (1, 6) days, with 57.25% of patients being admitted within 3 days after the onset of AP. Fourth, we did not define the changes in ECG and echocardiography in these patients. Several studies have noted that abnormal changes in the ECG and echocardiogram may occur earlier than serological indicators during AP. Fifth, other CIs such as N-pro-BNP, troponin I and T, and serum hydroxybutyrate dehydrogenase were also related to adverse clinical outcomes in AP (13–15). However, due to the limitations of the conditions, this study did not collect these data from the subjects.

In conclusion, our study demonstrates that elevated CK-MB levels (>24 U/ml) are an independent risk factor for adverse clinical outcomes in AP. Further attention should be given to AP-associated cardiac injury in the future.

## Data availability statement

The original contributions presented in the study are included in the article/Supplementary material, further inquiries can be directed to the corresponding author.

## Ethics statement

Written informed consent was obtained from the individual(s) for the publication of any potentially identifiable images or data included in this article.

## Author contributions

YZ: Funding acquisition, Project administration, Writing—review and editing. X-TJ: Writing—original draft. LD: Writing—original draft. XH: Writing—review and editing. Y-PL: Methodology, Writing—review and editing. H-JK: Methodology, Writing—review and editing. H-FX: Supervision, Writing—review and editing. L-YL: Data curation, Writing—review and editing. W-HH: Validation, Writing—review and editing. LX: Data curation, Writing—review and editing. N-HL: Project administration, Resources, Writing—review and editing.
