# Supplementary material for: Elevated CK-MB levels are associated with adverse clinical outcomes in acute pancreatitis: a propensity score-matched study
Source: Front Med (Lausanne). 2023 Sep 8;10:1256804. doi: 10.3389/fmed.2023.1256804 (PMC10514671; doi:10.3389/fmed.2023.1256804)
Supplement: Supplementary file 1 [file Data_Sheet_1.docx]

**Table S1**. Univariate analysis of patients’ clinical outcomes, and health care utilization between patients with elevated myocardial enzyme levels and normal myocardial enzyme levels among all 4802 patients.

|  | Normal group | Elevated group | P |
| --- | --- | --- | --- |
| **CK-MB**(ref: ≤24 U/mL),n (%) | 3906(81.34） | 896(18.66） |  |
| SAP,n (%) | 699(17.90) | 408(45.55） | <0.001 |
| IPN, n (%) | 239(6.12） | 120(13.39） | <0.001 |
| Mortality, n (%) | 155(3.97） | 148(16.52） | <0.001 |
| Length of hospital stay, days | 8(5.00,13.00） | 11(7.00,21.00） | <0.001 |
| Admission of ICU , n (%) | 672(17.20） | 439(49.00） | <0.001 |
| Total hospitalization charges, thousand yuan | 19.18(10.04,35.76） | 33.03(14.97,90.16） | <0.001 |
| **AST** (ref: ≤35 U/mL),n (%) | 2648(55.14） | 2154(44.86） |  |
| SAP,n (%) | 490(18.50） | 617(28.64） | <0.001 |
| IPN, n (%) | 169(6.38） | 190(8.82） | 0.001 |
| Mortality, n (%) | 98(3.70） | 205(9.52） | <0.001 |
| Length of hospital stay, days | 8(5.00,13.00） | 9(6.00,15.00） | <0.001 |
| Admission of ICU , n (%) | 461(17.41） | 650(30.18） | <0.001 |
| Total hospitalization charges, thousand yuan | 16.41(9.18,33.27） | 26.76(13.67,50.51） | <0.001 |
| **LDH**(ref: ≤250 U/mL),n (%) | 1423(29.63） | 3379(70.37） |  |
| SAP,n (%) | 101(7.10） | 1006(29.77） | <0.001 |
| IPN, n (%) | 60 (4.22） | 299(8.85） | <0.001 |
| Mortality, n (%) | 25 (1.76） | 278(8.23） | <0.001 |
| Length of hospital stay, days | 7 (4.00,10.00） | 9(6.00,15.00） | <0.001 |
| Admission of ICU , n (%) | 650(30.18） | 1027(30.39） | <0.001 |
| Total hospitalization charges, thousand yuan | 13.68(7.94,26.66） | 24.98(12.59,48.00） | <0.001 |
| **CK** (ref: ≤200 U/mL),n (%) | 4187(87.19） | 615(12.81） |  |
| SAP,n (%) | 783(18.70） | 324 52.68 | <0.001 |
| IPN, n (%) | 253(6.04） | 106 (17.24) | <0.001 |
| Mortality, n (%) | 175(4.18） | 128（20.81） | <0.001 |
| Length of hospital stay, days | 8(5.00,13.00） | 12(7.00,26.00） | <0.001 |
| Admission of ICU , n (%) | 778(18.58） | 333(54.15) | <0.001 |
| Total hospitalization charges, thousand yuan | 19.57(10.19,36.31） | 41.66(16.94,116.88） | <0.001 |

AST,aspartate transaminase;LDH,lactatede hydrogenase;CK,cretinekinase;

CK-MB,Creatine Kinase Myocardial Band; SAP,severe acute pancreatitis; IPN,infected pancreatic necrosis;ICU,intensive careunit.Quantitative variables are presented as medians (interquartile ranges) and were analyzed using the Mann-Whitney U test. Categorical variables are reported as absolute numbers and proportions, and were tested by the chi-square test

**Table S2.** Univariate analysis for the association of patients’ demographic characteristics, aetiologies, and comorbid conditions with the risk of mortality among all 4802 patients.

|  | Mortality (n=303) | Survival (n=4499) | OR (95% CI) | P value |
| --- | --- | --- | --- | --- |
| Sex (ref: female) |  |  |  |  |
| Male | 183(60.40%) | 2806(62.37%) | 1.09(0.86-1.38) | 0.493 |
| Age, (ref: ≤53.5yr) |  |  |  |  |
| >53.5 | 186(61.39%) | 1717(38.16%) | 2.58 (2.03-3.27) | <0.001 |
| BMI, (ref: ≤24 kg/m^2^) |  |  |  |  |
| >24 | 142(46.86%) | 2114(46.99%) | 1.00(0.79-1.26) | 0.967 |
| Admission within 72h after AP onset | 173(57.10%) | 2594(57.66%) | 1.02(0.81-1.30) | 0.848 |
| Aetiology (ref: biliary) |  |  |  |  |
| Biliary | 167(55.12%) | 2017(44.83%) | - | - |
| Alcoholic | 21(6.93%) | 237(5.27%) | 1.07(0.67-1.72) | 0.779 |
| Hyperlipidemia | 61(20.13%) | 1422(31.61%) | 0.52(0.38-0.70) | <0.001 |
| Mixed | 23(7.59%) | 264(5.87%) | 1.05(0.67-1.66) | 0.826 |
| Others | 31(10.23%) | 559(12.42%) | 0.67(0.45-0.99) | 0.047 |
| Smoking history (ref: no) |  |  |  |  |
| Yes | 91(30.03%) | 1253(27.85%) | 1.11(0.86-1.43) | 0.413 |
| Alcohol history (ref: no) |  |  |  |  |
| Yes | 104(34.32%) | 1305(29.01%) | 1.28(1.00-1.64) | 0.050 |
| Hypertension (ref: no) |  |  |  |  |
| Yes | 86(28.38%) | 785(17.45%) | 1.88(1.44-2.44) | <0.001 |
| Diabetes mellitus (ref: no) |  |  |  |  |
| Yes | 35(11.55%) | 547(12.16%) | 1.06(0.74-1.52) | 0.754 |
| CK-MB (ref: ≤24 U/mL) |  |  |  |  |
| >24 | 148(48.84%) | 748(16.63%) | 4.79(3.77-6.08) | <0.001 |
| CK (ref: ≤200 U/mL) |  |  |  |  |
| >200 | 128(42.24%) | 487(10.82%) | 6.03(4.71-7.71) | <0.001 |
| LDH (ref: ≤250U/mL) |  |  |  |  |
| >250 | 278(91.75%) | 3101(68.93%) | 5.01(3.31-7.59) | <0.001 |
| AST (ref: ≤35 U/mL) |  |  |  |  |
| >35 | 205(67.66%) | 1949(43.32%) | 2.74(2.14-3.51) | <0.001 |

**Table S3**. Univariate analysis for the association of patients’ demographic characteristics, aetiologies, and comorbid conditions with the risk of SAP among all 4802 patients.

|  | SAP  (n=1107) | Non-SAP (n=3695) | OR (95% CI) | P value |
| --- | --- | --- | --- | --- |
| Sex (ref: female) |  |  |  |  |
| Male | 707(63.87%) | 2282(61.76) | 1.09(0.95 -1.26) | 0.205 |
| Age, (ref: ≤53.5yr) |  |  |  |  |
| >53.5 | 493(44.53%) | 1410(38.16%) | 1.30 (1.14 -1.49) | <0.001 |
| BMI, (ref: ≤24 kg/m^2^) |  |  |  |  |
| >24 | 594(53.66%) | 1662(44.98%) | 1.42(1.24-1.62) | <0.001 |
| Admission within 72h after AP onset | 646(58.36%) | 2121(57.4%) | 1.04(0.91-1.19) | 0.573 |
| Aetiology (ref: biliary) |  |  |  |  |
| Biliary | 489(44.17%) | 1695(45.87%) | - | - |
| Alcoholic | 66(5.96%) | 192(5.20%) | 1.19(0.89-1.60) | 0.248 |
| Hyperlipidemia | 348(31.44%) | 1135(30.71%) | 1.06 (0.91-1.24) | 0.446 |
| Mixed | 102(9.21%) | 185(5.01%) | 1.91(1.47-2.48) | <0.001 |
| Others | 102(9.21%) | 488(13.21%) | 0.73 (0.57- 0.92) | 0.007 |
| Smoking history (ref: no) |  |  |  |  |
| Yes | 336 (30.35%) | 1008(27.28%) | 1.16(1.00-1.35) | 0.046 |
| Alcohol history (ref: no) |  |  |  |  |
| Yes | 382(34.51%) | 1027( 27.79%) | 1.37(1.19-1.58) | <0.001 |
| Hypertension (ref: no) |  |  |  |  |
| Yes | 273(24.66%) | 598 (16.18%) | 1.70(1.44-1.99) | <0.001 |
| Diabetes mellitus (ref: no) |  |  |  |  |
| Yes | 154(13.91%) | 428 (11.58%) | 1.23(1.01-1.50) | 0.038 |
| CK-MB (ref: ≤24 U/mL) |  |  |  |  |
| >24 | 408(36.86%) | 488(13.21%) | 3.84(3.29-4.48) | <0.001 |
| CK (ref: ≤200 U/mL) |  |  |  |  |
| >200 | 324(29.27%) | 291(7.88%) | 4.84(4.06-5.77) | <0.001 |
| LDH (ref: ≤250U/mL) |  |  |  |  |
| >250 | 1006(90.88%) | 2373 (64.22%) | 5.55(4.48-6.88) | <0.001 |
| AST (ref: ≤35 U/mL) |  |  |  |  |
| >35 | 617(55.74%) | 1537 (41.60%) | 1.77(1.54-2.02) | <0.001 |

**Table S4.** Univariate analysis for the association of patients’ demographic characteristics, aetiologies, and comorbid conditions with the risk of IPN among all 4802 patients.

|  | INP  (n=359) | Non-INP (n=4443) | OR (95% CI) | P value |
| --- | --- | --- | --- | --- |
| Sex (ref: female) |  |  |  |  |
| Male | 218(60.72%) | 2771(62.37%) | 1.07(0.86-1.33) | 0.537 |
| Age, (ref: ≤53.5yr) |  |  |  |  |
| >53.5 | 131(36.49%) | 1772(39.88%) | 1.16(0.92 1.44) | 0.206 |
| BMI, (ref: ≤24 kg/m^2^) |  |  |  |  |
| >24 | 162(45.13%) | 2094(47.13%) | 1.08(0.87-1.35) | 0.464 |
| Admission within 72h after AP onset | 142(39.55%) | 2625(59.08%) | 2.21(1.77,2.75) | <0.001 |
| Aetiology (ref: biliary) |  |  |  |  |
| Biliary | 156(43.45%) | 2028(45.64%) | - | - |
| Alcoholic | 19 (5.29%) | 239(5.38%) | 1.03 (0.63-1.70) | 0.896 |
| Hyperlipidemia | 104(28.97%) | 1379(31.04%) | 0.98(0.76-1.27) | 0.880 |
| Mixed | 24(6.69%) | 263(5.92%) | 1.19 (0.76-1.86) | 0.455 |
| Others | 56(15.60%) | 534(12.02%) | 1.36 (0.99-1.88) | 0.058 |
| Smoking history (ref: no) |  |  |  |  |
| Yes | 95(26.46%) | 1249(28.11%) | 1.09(0.85-1.39) | 0.503 |
| Alcohol history (ref: no) |  |  |  |  |
| Yes | 104(28.97%) | 1305(29.37%) | 1.02(0.81-1.29) | 0.872 |
| Hypertension (ref: no) |  |  |  |  |
| Yes | 80(22.28%) | 791(17.80%) | 1.32(1.02-1.72) | 0.035 |
| Diabetes mellitus (ref: no) |  |  |  |  |
| Yes | 53(14.76%) | 529(11.91%) | 1.28(0.94-1.74) | 0.111 |
| CK-MB (ref: ≤24 U/mL) |  |  |  |  |
| >24 | 120(33.43%) | 776(17.47%) | 2.37(1.88-2.99) | <0.001 |
| CK (ref: ≤200 U/mL) |  |  |  |  |
| >200 | 106(29.53%) | 509(11.46%) | 3.24(2.54-4.14) | <0.001 |
| LDH (ref: ≤250U/mL) |  |  |  |  |
| >250 | 299(83.29%) | 3080(69.32%) | 2.21(1.66-2.93) | <0.001 |
| AST (ref: ≤35 U/mL) |  |  |  |  |
| >35 | 190(52.92%) | 1964(44.20%) | 1.42(1.14-1.76) | <0.001 |

**Table S5.** Baseline characteristics of 2767 / 2035 patients of Admission within 72h / after 72h after AP onset

| Characteristics | CK-MB（within 72h） | |  | CK-MB（after 72h） | |  |
| --- | --- | --- | --- | --- | --- | --- |
|  | Elevated CK-MB  (n=653) | Normal CK-MB  (n=2114) | P | Elevated CK-MB  (n=243) | Normal CK-MB  (n=1792) | P |
| Male, n (%) | 458 (70.14) | 1327 (62.77) | 0.001 | 154 (63.37) | 1050 (58.59) | 0.155 |
| Age, years | 45 (35.00,57.00) | 48 (36.00,62.00) | 0.009 | 51(40,67) | 51 (40,63) | 0.371 |
| BMI, kg/m^2^ | 24.49(22.68,27.55) | 24.03(22.02,26.15) | <0.001 | 23.81 (21.26,25.22) | 23.11 (20.77,25.21) | 0.027 |
| Admission  time after AP | 2(1.00,2.00) | 2(1.00,3.00) | <0.001 | 7(5.00,14.00) | 7(5.00,15.00) | 0.784 |
| onset, days |  |  |  |  |  |  |
| Etiology, n (%) |  |  | <0.001 |  |  | 0.167 |
| Biliary | 199(30.47) | 894(42.29) |  | 115(47.33) | 976(54.46) |  |
| Alcoholic | 37 (5.67) | 115 (5.44) |  | 12 (4.94) | 94(5.25) |  |
| Hyperlipidemia | 318(48.70) | 759(37.61) |  | 61(25.10) | 345(19.25) |  |
| Others | 42 (6.43) | 226(10.69) |  | 39 (16.05) | 283(15.79) |  |
| Mixed | 57 (8.73) | 120 (5.68) |  | 16 (6.58) | 94(5.25) |  |
| Smoking history, n (%) | 224 (34.30) | 586 (27.72) | 0.001 | 78 (32.10) | 456 (25.45) | 0.027 |
| Drinking history, n (%) | 234 (35.83) | 616 (29.14) | 0.001 | 83 (34.16) | 476 (26.56) | 0.013 |
| Hypertension, n (%) | 127 (19.45) | 378 (17.88) | 0.365 | 61 (25.10) | 305 (17.02) | 0.002 |
| Diabetes mellitus, n (%) | 108 (16.54) | 240 (11.35) | <0.001 | 27 (11.11) | 207(11.55) | 0.840 |

**Table S6.** Univariate analysis of patients’ clinical outcomes, and health care utilization between 2767/2035 patients of Admission within 72h / after 72h after AP onset

| Characteristics | CK-MB(within 72h) | |  | CK-MB(after 72h) | |  |
| --- | --- | --- | --- | --- | --- | --- |
|  | Elevated CK-MB  (n=653) | Normal CK-MB  (n=2114) | P | Elevated  CK-MB  (n=243) | Normal  CK-MB  (n=1792) | P |
| SAP, n (%) | 307(47.01) | 339(16.04) | <0.001 | 101(41.56) | 360(20.09) | <0.001 |
| IPN, n (%) | 84(12.86) | 58(2.74) | <0.001 | 36(14.81) | 181(10.10) | 0.025 |
| Mortality, n (%) | 102(15.62) | 71(3.36) | <0.001 | 46(18.93) | 84(4.69) | <0.001 |
| Length of hospital stay, days | 11(7.00,21.00) | 7(5.00,12.00) | <0.001 | 10(6.00,21.00) | 8(6.00,14.00) | <0.001 |
| Admission of ICU , n (%) | 329(50.38) | 346(16.37) | <0.001 | 110(45.27) | 326 (18.19) | <0.001 |
| Total hospitalization charges, thousand yuan | 33.51(15.12,  89.98) | 16.73(9.58,  31.97) | <0.001 | 31.10(14.45,  91.18) | 22.23(10.84,  41.04) | <0.001 |
